# Evaluation of Multi-Scale Climate Effects on Annual Recruitment Levels of the Japanese Eel, *Anguilla japonica*, to Taiwan

**DOI:** 10.1371/journal.pone.0030805

**Published:** 2012-02-23

**Authors:** Wann-Nian Tzeng, Yu-Heng Tseng, Yu-San Han, Chih-Chieh Hsu, Chih-Wei Chang, Emanuele Di Lorenzo, Chih-hao Hsieh

**Affiliations:** 1 Department of Life Science and Institute of Fisheries Science, National Taiwan University, Taipei, Taiwan; 2 Department of Environmental Biology and Fisheries Science, National Taiwan Ocean University, Keelung, Taiwan; 3 Department of Atmospheric Sciences, National Taiwan University, Taipei, Taiwan; 4 Earth Dynamic Center, National Cheng-Kung University, Tainan, Taiwan; 5 Department of Geography, National Changhua University of Education, Changhua, Taiwan; 6 National Museum of Marine Biology and Aquarium, Checheng, Pingtung, Taiwan; 7 School of Earth and Atmospheric Sciences, Georgia Institute of Technology, Atlanta, Georgia, United States of America; 8 Institute of Oceanography, National Taiwan University, Taipei, Taiwan; 9 Institute of Ecology and Evolutionary Biology, National Taiwan University, Taipei, Taiwan; National Oceanic and Atmospheric Administration/National Marine Fisheries Service/Southwest Fisheries Science Center, United States of America

## Abstract

Long-term (1967–2008) glass eel catches were used to investigate climatic effects on the annual recruitment of Japanese eel to Taiwan. Specifically, three prevailing hypotheses that potentially explain the annual recruitment were evaluated. Hypothesis 1: high precipitation shifts the salinity front northward, resulting in favorable spawning locations. Hypothesis 2: a southward shift of the position of the North Equatorial Current (NEC) bifurcation provides a favorable larval transport route. Hypothesis 3: ocean conditions (eddy activities and productivity) along the larval migration route influence larval survival. Results of time series regression and wavelet analyses suggest that Hypothesis 1 is not supported, as the glass eel catches exhibited a negative relationship with precipitation. Hypothesis 2 is plausible. However, the catches are correlated with the NEC bifurcation with a one-year lag. Considering the time needed for larval transport (only four to six months), the one-year lag correlation does not support the direct transport hypothesis. Hypothesis 3 is supported indirectly by the results. Significant correlations were found between catches and climate indices that affect ocean productivity and eddy activities, such as the Quasi Biennial Oscillation (QBO), North Pacific Gyre Oscillation (NPGO), Pacific Decadal Oscillation (PDO), and Western Pacific Oscillation (WPO). Wavelet analysis reveals three periodicities of eel catches: 2.7, 5.4, and 10.3 years. The interannual coherence with QBO and the Niño 3.4 region suggests that the shorter-term climate variability is modulated zonally by equatorial dynamics. The low-frequency coherence with WPO, PDO, and NPGO demonstrates the decadal modulation of meridional teleconnection via ocean–atmosphere interactions. Furthermore, WPO and QBO are linked to solar activities. These results imply that the Japanese eel recruitment may be influenced by multi-timescale climate variability. Our findings call for investigation of extra-tropical ocean dynamics that affect survival of eels during transport, in addition to the existing efforts to study the equatorial system.

## Introduction

Climatic effects on fluctuations of fish populations and fisheries have long been recognized [1] and continue to be critical: understanding these effects is an essential step toward conserving and managing marine resources [2], [3], [4]. The most widely studied climatic forcing impacts on fishes include those at an interannual scale, such as El Niño/Southern Oscillation (ENSO) [5], [6], and at a decadal scale, such as Pacific Decadal Oscillation [7], [8], North Pacific Gyre Oscillation [9], and North Atlantic Oscillation [10], [11]. In eastern Asia, commercial fish species are also found to be influenced by climate [12], [13], [14]. The fluctuation of the Japanese eel, *Anguilla japonica*, has gained particular attention [15], due to its high economic value [16], complex life history [17], and its declining recruitment since the 1970s [18], [19]. A similar declining trend has also been reported for the European eel, *A. anguilla*, and American eel, *A. rostrata* [20]. The reason for the declines in recruitment of these temperate *Anguilla* eels is not clear, but is possibly caused by overfishing, habitat degradation, pollutions, parasites, virus, and global climate change [19], [21], [22], [23], [24], [25], [26]. In addition to the trend for a long-term decline in Japanese eel, fluctuations at interannul and decadal scales are also observed [19], [21], [24], which warrant further investigation.

The Japanese eel is a catadromous fish, widely distributed in the western Pacific, from the Philippines in the south, through Taiwan, mainland China, Korea, to Japan in the north [27]. The Japanese eel spawns in the waters west of the Mariana Islands, near 14°–16°N, 134°–143°E, between April and August [28], [29], [30]. After hatching, the eel larvae, called leptocephali, drift with the westward North Equatorial Current (NEC) and then the northward Kuroshio Current towards the continental shelf, where they metamorphose into glass eels, becoming pigmented elvers in the estuaries [17], [31]. The passive migration from the spawning area to the estuaries of Taiwan takes approximately four to six months [31]. After living in freshwater for five to ten years [32], [33], the yellow eels become silver eels and return to the spawning area to spawn and finish their life cycle; however, the exact return route is still unknown [17].

It has been suggested that recruitment variability of the Japanese eel is affected by ocean–atmospheric forcing [15]. In particular, the latitudinal shifts of spawning locations in relation to larval transport by the NEC are considered to be an important determinant of recruitment success [13]. If the eels can travel westward using the NEC and enter the Kuroshio Current, they have a greatly enhanced probability of recruitment success. By contrast, if they are entrained into the south-flowing Mindanao Current or mesoscale eddies east of Taiwan, recruitment is reduced [34]. Specifically, when precipitation is low during some ENSO years, the salinity front (and thus the spawning location) may move considerably southward, therefore increasing the possibility that the eel larvae will enter the Mindanao Current [13], [35]. In addition, the bifurcation latitude of the NEC varies both seasonally and interannually [36], which potentially also affects the recruitment variability of the Japanese eel [37]. In particular, ENSO events shift the bifurcation latitude of NEC northward, which results in more NEC water flowing into the Mindanao Current, and hampers eel recruitment. [37]. Nevertheless, these hypotheses about eel recruitment success have mainly been formulated based on particle-tracking simulation models and limited observations. Yet another possible climatic effect is the change in ocean productivity that may be critical for feeding success and survival of larvae during their migration route [15], [24]. Climatic factors (e.g. Pacific Decadal Oscillation, PDO) have been suggested as important [15], but not investigated for the Japanese eel.

While it is speculated that climate variability might have crucial impacts on the Japanese eel recruitment, direct comparisons between the long-term data for both recruitment and climate are scarce. In our study, we took advantage of the unique long-term (1967–2008) record of glass eels caught in the estuaries of Taiwan, where the earliest catches in eastern Asia occur, to investigate multi-timescale climatic influences on the annual recruitment of the Japanese eel. We evaluated three prevailing hypotheses used to explain the annual Japanese eel recruitment [15]. Hypothesis 1: high precipitation shifts the salinity front northward, resulting in favorable spawning locations (“Spawning location hypothesis”). Hypothesis 2: a southward shift of the NEC bifurcation location provides favorable larval transport route (“Larval transport hypothesis”). Hypothesis 3: ocean conditions (such as eddy activities and productivity) along the larval migration route influence larval survival (“Ocean condition hypothesis”). To test Hypothesis 1, we examined precipitation around the eel spawning area. To test Hypothesis 2, we defined the latitudinal shift of the NEC bifurcation location by combining observational and modeling data. For Hypothesis 3, we investigated various climate indices that have been shown as likely to affect ocean productivity and/or eddy activities, such as the Southern Oscillation Index (SOI), Quasi Biennial Oscillation (QBO), North Pacific Gyre Oscillation (NPGO), North Pacific Index (NPI), Pacific Decadal Oscillation (PDO), and Western Pacific Oscillation (WPO). In addition, accumulating evidence suggests that solar activities may have significant effects on climate ([38] and references therein); thus, we also included the number of sunspots in our analyses.

## Materials and Methods

### Annual catches of glass eels as a proxy for recruitment

**Figure 1:**
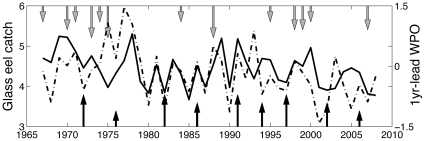
Time series of log_10_(eel catch) (bold line) and 1-year leading summer WPO index (dashed line). The black arrows represent the El Nino years, and the gray arrows represent La Nina years. The length of arrow indicates the strength of the events. The glass eel catches were significantly correlated with summer WPO with 1-year lag. The responses of eel catches to El Nino events were not clear.

Data for the annual glass eel catch of the Japanese eel in the estuaries of Taiwan from 1967 to 2008 were compiled from monthly reports in the Taiwan Fisheries Yearbook (Fisheries Agency, Council of Agriculture, Executive Yuan), which was collected daily by the district Fisheries Association of Taiwan (only quarterly data were available from 2006). Glass eels caught in the estuaries during their upstream migration in winter are the sole source for aquaculture, because artificial propagation techniques have not yet reached a commercially viable scale [39]. Due to the high economic value, the fishing effort for glass eels is very high [40]. Unfortunately, the glass eel fishery was unregulated and fishing effort unreported; therefore, the catch per unit effort (CPUE) data were not available. As the fishing efforts are substantially high, the catches of glass eels may be representative of Japanese eel recruitment, similar to those used in other studies [24], [35]. The annual recruitment of the Japanese eel in Taiwan was calculated from July in one year to June of the following year, because the recruitment season for glass eels in Taiwan occurs mainly from October to April, and peaks between December and February [41], [42]. The glass eels caught during this time interval were considered to be of the same annual cohort [21] in our time series analyses. This time series represents the longest annual recruitment index of Japanese eel to Taiwan (Figure 1). As no CPUE data for the Japanese eel exist, this glass eel catch data is the best proxy for the annual recruitment of Japanese eel available in the world.

### Precipitation around the eel spawning area

To investigate hypothesis 1 (shifting spawning location due to precipitation strength), monthly precipitation data around the eel spawning area (14°–16°N, 134°–143°E) were extracted from the monthly mean NCEP-NCAR reanalysis I product [43] since 1950. The monthly precipitation data were spatially averaged in order to obtain a unique time series, which compared well with that derived from the GPCP observation [44] after 1979. Such a similarity indicates that the NCEP precipitation data in this area should be reliable.

### Analyses of latitudinal shifts of the NEC bifurcation

As one of the main objectives of this study is to examine the relationship between the latitudinal shift of the NEC bifurcation and the annual recruitment of Japanese eel, we need to be clear how we defined the NEC bifurcation location. The long-term latitudinal variation of the NEC bifurcation location was estimated from the sea surface height (SSH) data of a high-resolution global ocean circulation model, and validated using satellite altimetry data.

This high-resolution Ocean General Circulation Model for the Earth Simulator (OFES) was developed by the Earth Simulator Center, Japan Agency for Marine-Earth Science and Technology, and used to hindcast the sea level variability. This OFES is based on the Modular Ocean Model (MOM3), while the model domain covers a near-global region extending from 75°S to 75°N with a horizontal grid spacing of 0.1°. We analyzed two model simulations with different surface forcings. The first simulation was driven by the daily mean wind stress from the NCEP-NCAR reanalysis I from 1950 to 2007, and the freshwater flux calculated from precipitation–evaporation rates through the same reanalyzed data [45]. Following Wang *et al.* [46], the bifurcation latitude of the NEC was calculated using Empirical Orthogonal Function (EOF) analysis of the detrended sea level variability data for the area of 8°–13°N and 120°–140°E. Since sea surface variability is sensitive to surface wind curl, we further investigated the other model simulation, which is driven by the realistic QuikSCAT wind from July 1999 to 2007 [47].

**Figure 2:**
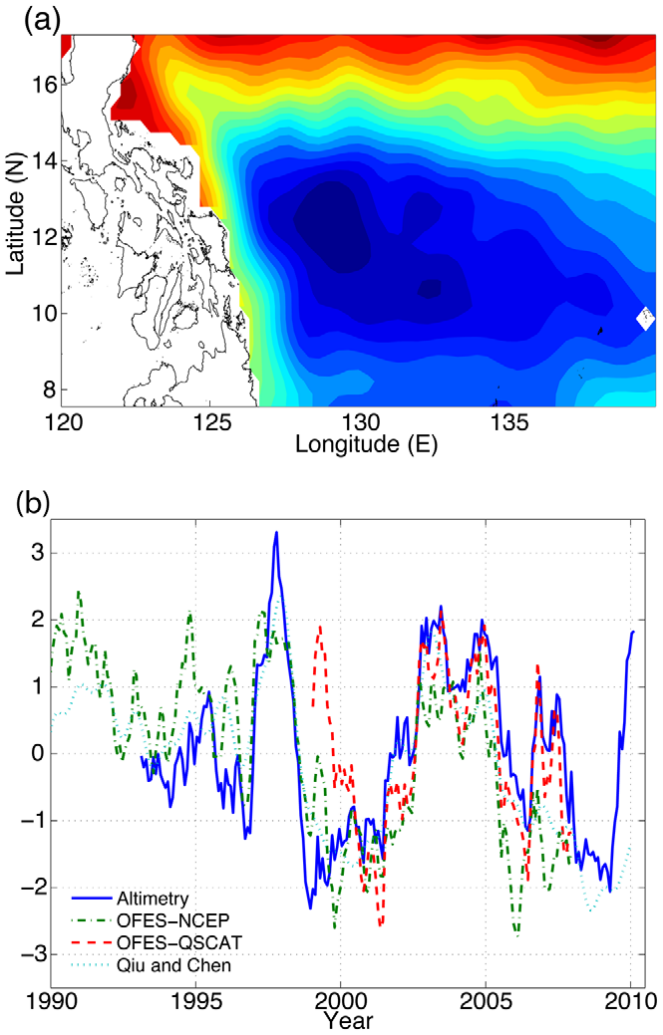
Index for latitudinal shift of NEC bifurcation. In (a), the contour illustrates the first EOF mode of the area east of Philippines from the altimetry data. In (b), time series represent the normalized PC1 for the altimetry data and the OFES model results forced by NCEP and QSCAT wind, respectively. The normalized NEC bifurcation latitude determined by Qiu and Chen (2010b) is also shown. The time series from four calculations show strong coherence.

Furthermore, the model results were carefully validated with the satellite altimetry data from 1993 to 2010. The altimetry from the Map of Absolute Dynamic Topography was produced by Ssalto/Duacs and distributed by Aviso with support from CNES, based on satellites Topex/Poseidon, Jason-1, ERS-1/2 and Envisat. These altimetry data contain near-real time and delayed time products. We use the delayed time product with “ref” version in this study. The bifurcation latitude of NEC was also calculated using the same EOF analysis of the detrended altimetry data for the same area as the OFES results. We used the first EOF1 (accounting for 62.36% of the total variance, Figure 2a) to represent variation in the NEC bifurcation location. As Figure 2a illustrates, the maximum magnitude occurred at around 12–13°N and reduced gradually southward and northward, indicating the main axis of the NEC.

The latitudinal shifts of NEC bifurcation calculated from the three different analyses (sea surface variability from OFES driven by NCEP-NCAR daily wind and QuikSCAT wind, as well as satellite altimetry) exhibited a similar pattern (Figure 2b). This pattern is also consistent with the NEC bifurcation latitude described by Qiu and Chen [48]. Note that the sea surface variability of OFES driven by the QuikSCAT wind more closely resembles the variability in altimetry than that driven by NCEP-NCAR wind. However, the QuikSCAT wind is only available after 1999, and the OFES driven by QuikSCAT requires some spin-up transient, and its behavior may therefore not be very stable during the first year or so [47]. In the following analyses with respect to eels, we used the EOF1 of the OFES simulations driven by NCEP-NCAR as the proxy for latitudinal shifts of NEC bifurcation, because this is the only time series extending back to 1960 (comparable to the glass eel catch data).

### Climate indices

In addition to NEC, we investigated various climate indices that could potentially affect eel dynamics. For tropical climate signals, we investigated ENSO related indices (Table 1) and the QBO. The QBO represents oscillation of the equatorial zonal wind between easterlies and westerlies, with an average period of 28 months [49]. The QBO explains the largest fraction of the circulation variability in the middle atmosphere [50]. For the extra-tropics, we examined several important indexes, such as PDO (the leading EOF of North Pacific monthly sea surface temperature variability poleward of 20°N) [7], NPI (the area-weighted sea level pressure over the region 30°N–65°N, 160°E–140°W) [51], and NPGO (the second EOF of sea surface height anomalies in the North Pacific) [52]. These mid-latitude climate indices have been shown to affect North Pacific ecosystem dynamics [1], [8], [52]. We also investigated the WPO (the second EOF of 500 mb geopotential height), which has been shown to affect ocean dynamics in the Pacific through teleconnection [53], [54], [55]. Moreover, WPO was found to be closely related with eddy kinetic energy fields in the subtropical region east of Taiwan [56]. The time series of eddy kinetic energy from 1992 onward [56] was also included in our analyses. We further examined solar activities (sunspot number), as accumulating evidence suggests that solar activities may affect the Pacific climate system [38], [57], [58].

**Table 1:** Results of regression analyses of log_10_(eel catch) against climate indices.

	Annual	Spring	Summer	Autumn	Winter
Precipitation	NS	NS	NS	NS	−0.316(1 yr)
NEC	−0.310(3 yr)	NS	−0.343(3 yr)	−0.282(1 yr)	NS
Niño3.4	NS	NS	NS	NS	NS
Niño4	NS	NS	NS	NS	NS
Niño3	NS	NS	NS	NS	NS
Niño1.2	NS	NS	NS	NS	NS
SOI	NS	NS	NS	NS	NS
QBO	−0.272(3 yr)	−0.339(1 yr) 0.394(2 yr)	−0.287(3 yr)	0.286(1 yr)	−0.320(0 yr)
NPGO	NS	NS	NS	NS	−0.334(1 yr)
PDO	−0.308(1 yr)	−0.399(2 yr)	NS	NS	−0.449(1 yr)
NPI	NS	NS	NS	NS	0.291(1 yr)
WPO	0.289(2 yr)	NS	0.472(1 yr)	0.417(1 yr)	NS
Eddy kinetic energy	NS	NS	NS	NS	NS
Sunspot	0.328(0 yr)	0.306(0 yr)	0.309(0 yr)	NS	0.367(0 yr)

Autocorrelation is accounted for using estimated generalized least squares. Data are normalized to unit mean and variance before analyses. When correlations are significant for multiple lags, all significant correlations are presented. The full table is provided in Supporting Information S1.

Only the regression coefficient significant at p<0.05 is presented. NS indicates that no significant correlation was found in all lags (0 to 5 years). Parenthesis encloses the years that the climate index leads the eel catches. We investigated the lag up to 5 years. For QBO, correlation beyond 3 years is omitted, considering its biennial nature.

### Long-term correlation between the annual eel recruitment and climate indices

We examined the influence of climate on the long-term variability of the annual eel recruitment, using regression analysis for each climate index. It should be noted that for each climate index, we analyzed four seasons as well as the annual mean. In our study, the year for climate indices started from spring, defined as March, April, and May, following the typical climatic seasonality. This leads to the definition of winter as December, and January and February of the following year. The lagged climate effects were tested up to five years. To account for serial dependence in time series data, the estimated generalized least squares (EGLS) method was used for hypothesis testing [59]. As this univariate analysis is used for exploring potential climate effects, the significant level is set as 5%, without correcting for multiple tests. Finally, we used stepwise multivariate regression to obtain the best-fit model. For those analyses, the eel catch data were log-transformed prior to analyses, in order to stabilize the variance. All time series were normalized to unit mean and variance prior to analyses.

### Multiscale analyses of climatic forcings using wavelet

The possible influence of particular climate patterns on the annual eel recruitment may not be stationary, and each climate pattern may affect the recruitment dynamics at a different scale. We therefore used wavelet analyses that require no assumption of stationarity and have the ability to determine the dominant modes of variability in frequency and how those modes vary over time [60], [61]. We used the Morlet wavelet function [60]. The 5% significance level was determined based on bootstrap simulations (1000 times), using the spectral synthetic test [62]. The spetral slope was obtained empirically from the time series data [62].

We then carried out cross-wavelet coherence and phase analyses to understand relationships between the environmental variables and eel catches. The wavelet coherency is defined as:
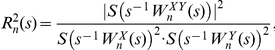
where *W* is the wavelet transform of the time series, *S* is a smoothing operator by running average [63]. The wavelet coherency phase is:
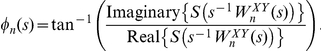
Both 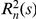 and 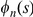 are functions of the time index *n* and the scale *s*. Similarly, the 5% significance level of wavelet cohereency was determined based on bootstrap simulations (1000 times), using the spectral synthetic test [62]. The spetral slope was obtained empirically from the time series data [62]. We did not apply wavelet analysis to eddy kinetic energy data, because the series was too short.

## Results

### Long-term relationships between glass eel catches and climate indices

Results of regressions between climate indices and glass eel catches in Taiwan suggested that climate variation might have affected the annual recruitment of Japanese eel to Taiwan (Table 1 and Supporting Information S1). Glass eel catches correlated negatively with winter precipitation around the eel spawning area, autumn NEC bifurcation, winter NPGO, and winter PDO with a one-year lag, and positively with winter NPI, and summer and autumn WPO with a one-year lag. Interestingly, sunspot numbers were also correlated with catches. The catches were correlated with QBO, and the complicated negative and positive correlations at lags were due to the quasi-biennial nature of QBO. The catches were only marginally (0.05<p<0.1, Supporting Information S1) correlated with ENSO. We further categorized El Nino and La Nina events into three levels of strength (based on the Oceanic Nino Index, ONI, http://ggweather.com/enso/oni.htm), but found that the effects of dominant ENSO events on catches were not consistent (Figure 1). For example, the low catches corresponded well to some strong ENSO events, such as the years of 1982–1983, 1986–1987 and 1997–1998. However, not all strong ENSO events corresponded to the low catch (e.g., 1992–1993). By contrast, fairly good correspondence was found between the catches and one-year leading summer WPO index (Figure 1). The best model of stepwise multivariate regression analysis included only the summer WPO and winter PDO in the predictors: Log_10_(eel catches) = 0.3*WPO–0.441*PDO (R^2^ = 0.277, p<0.001). This result suggested that the extra-tropic climate might play a role in affecting the Japanese eel. We further found a significant (however small) long-term declining trend on top of the fluctuations in catches (r = −0.367, p<0.05). As the declining trend is significant, we have also repeated all the analyses with detrended (with the linear trend removed) data and obtained qualitatively similar results.

### Multi-scale variation of annual eel recruitment in response to climate

**Figure 3:**
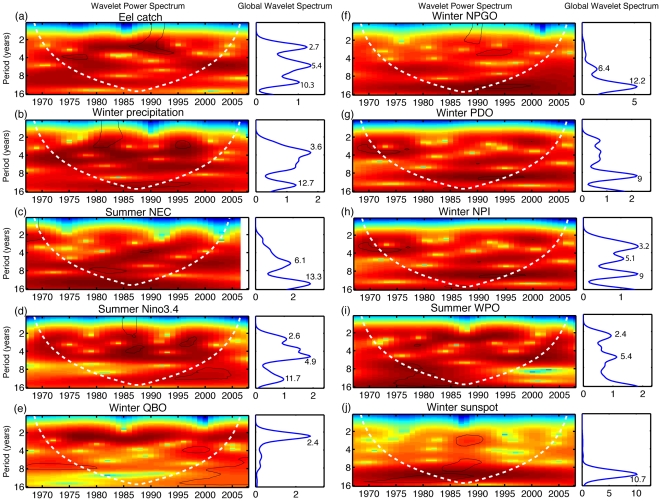
Results of wavelet analyses revealing the nonstationary fluctuations through time of the eel catch data as well as climate indices. The left panel represents the wavelet power spectrum and the right panel indicates the global power spectrum averaged over the time series for (a) log_10_(eel catch), (b) winter precipitation at the eel spawning area, (c) summer NEC bifurcation, (d) summer Niño3.4 index, (e) winter QBO index, (f) winter NPGO index, (g) winter PDO index, (h) winter NPI index, (i) summer WPO index, and (j) winter sunspot numbers. The vertical axis represents period (years). The local wavelet power spectrum provides a measure of the variance distribution of the time series according to time and for each periodicity; high variability is represented by red, whereas blue indicates a weak variability. The solid black contour encloses regions of greater than 95% confidence for a red-noise process with a lag-1 coefficient, and the white dashed line area indicates the cone of influence where edge effects become important. For the global power spectrum, periods corresponding to the peaks are indicated.

The results of wavelet analyses indicate that the periodicity of fluctuations changed through time for the eel catch time series as well as other climate indices (Figure 3). If we take the long-term average, we can roughly see three main periodicities of 2.7, 5.4 and 10.3 years in the time series of the eel catches (Figure 3a). The higher-frequency periodicities (2.7 and 5.4 years) are consistent with some parts of the periodicities of the Niño3.4, WPO, and perhaps QBO (Figures 3d, e, and i). The decadal-scale of the 10.3-year low-frequency periodicity appeared to agree with the 11-year solar cycle (Figure 3j). The variability of frequency changed over time, which revealed the nonstationary nature of eel catches and some climate indices (Figure 3). This led us to examine the cross wavelet coherence between eel catches and climate indices.

**Figure 4:**
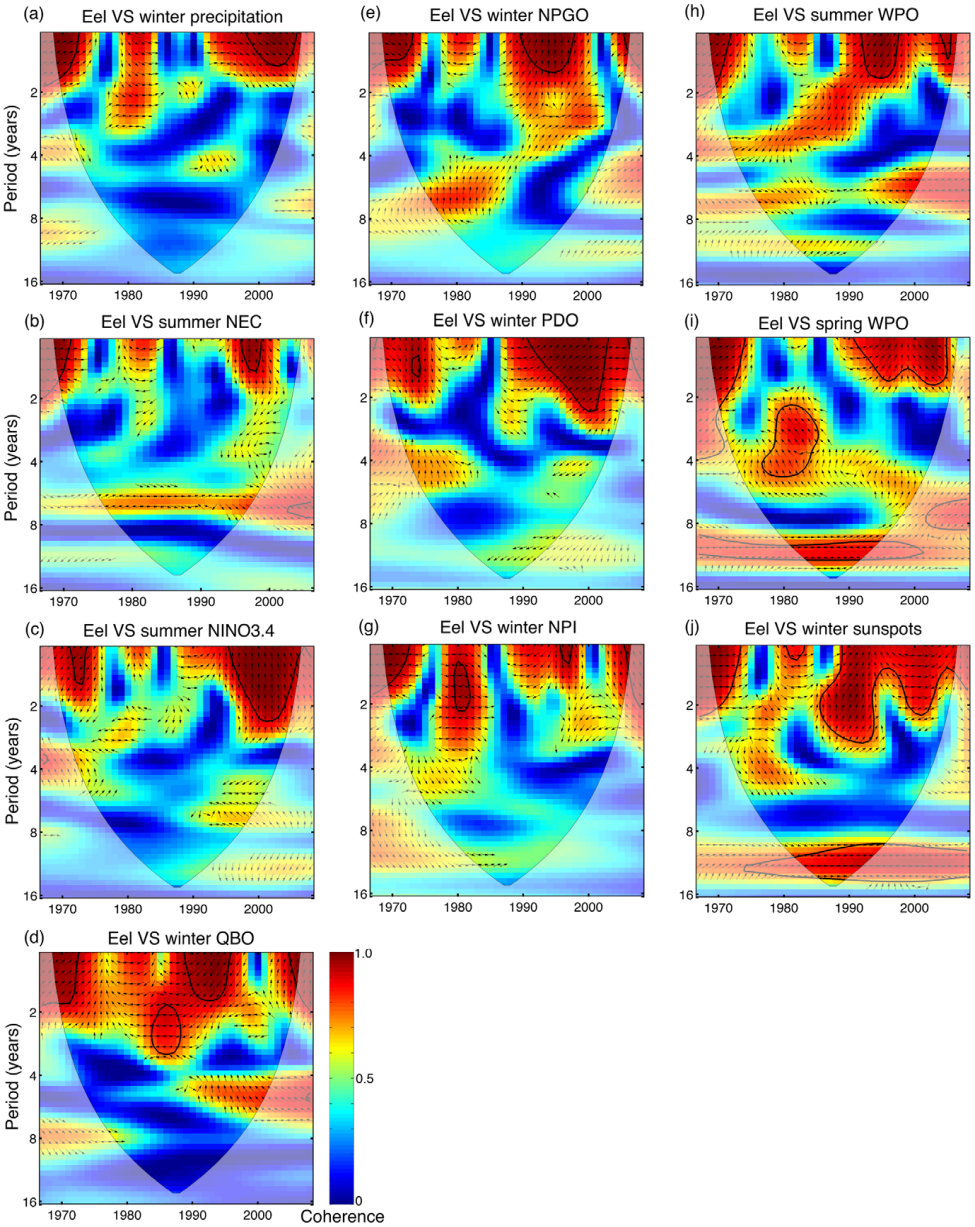
Cross wavelet coherence between log_10_(eel catch) and climate indexes: (a) winter precipitation at the eel spawning area, (b) summer NEC bifurcation, (c) summer Niño3.4, (d) winter QBO, (e) winter NPGO, (f) winter PDO, (g) winter NPI, (h) summer WPO, (i) spring WPO, and (j) winter sunspot numbers. The solid black contour encloses regions of greater than 95% confidence, and the shadowed area indicates the cone of influence. The phase relationship is shown as arrows, with in-phase pointing right, anti-face pointing left, and the environmental variable leading the eel catches by 90° pointing straight down.

The results of cross wavelet coherence analyses indicated more complicated relationships in addition to the long-term correlations in Table 1. The coherence of catches with winter precipitation, summer Niño3.4, winter NPI, and winter QBO occurs at the scale of one to three years (Figures 4a, c, d, and g). Coherence between catches and summer NEC bifurcation occurs at the time scale of five to seven years, although not statistically significant (Figure 4b). The coherence with winter NPGO, winter PDO, and summer WPO exists at various scales (Figures 4e, f, and h). Again, the coherence is not stationary. We further found a coherent relationship between catches versus spring WPO and winter sunspot numbers at the scale of nine to thirteen years (Figures 4i and j). These phenomena indicated that the annual recruitment of Japanese eel might have been affected by climate change at multiple time scales. (Note that we investigated wavelet coherence for four seasons, as well as in relation to the annual mean, and found either qualitatively similar results or less clear coherence than those presented in Figure 4.).

### Solar modulation of the Pacific climate and ocean–atmospheric interactions

**Figure 5:**
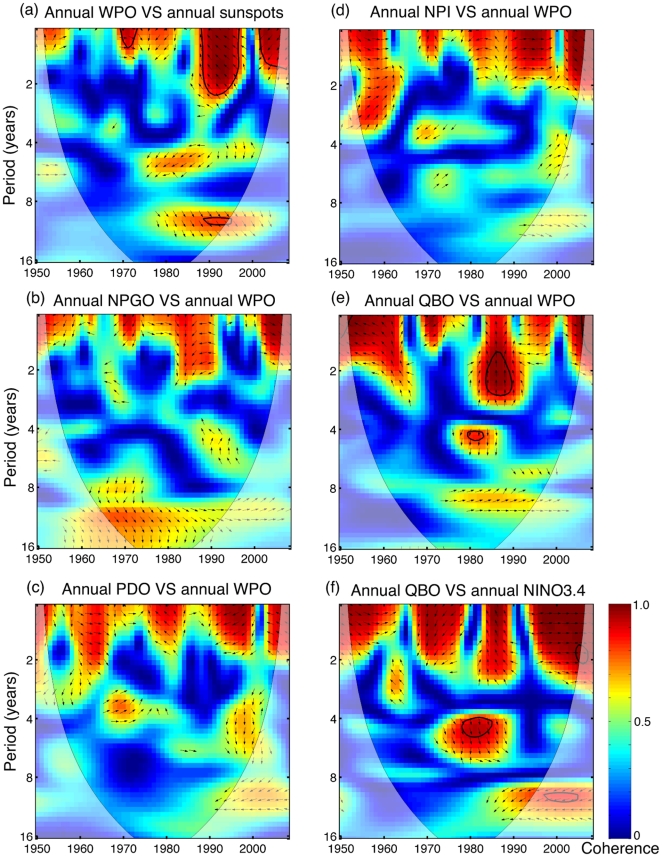
Cross wavelet coherence between climate indexes: (a) WPO versus sunspot numbers, (b) NPGO versus WPO, (c) PDO versus WPO, (d) NPI versus WPO, (e) QBO versus WPO, and (f) QBO versus Niño3.4. See Figure 4 for legends.

To further investigate North Pacific climate, we examined wavelet coherence between various climate indices that we identified as possibly affecting eels. We found coherence between sunspot numbers and WPO at the scale of nine to thirteen years (Figure 5a). However, the coherence has only existed since the late 1970s with a limited significant region. In addition to high-frequency (one to three years) coherence, WPO exhibited nonstationary (however, non-significant) correlation with NPGO and QBO at a low frequency of nine- to 13-year periodicity (Figures 5b and 5e). WPO seemed to show some coherence with PDO and NPI at a shorter period throughout the ∼60 years (since 1950) and started to show some additional low-frequency coherence after 1990 (Figures 5c and d). Stronger coherence at a low-frequency mode was found if analyses were done at specific seasons (Supporting Information S2). QBO showed nonstationary coherence with Niño3.4 (Figure 5f), a similar finding discussed in a review by Baldwin *et al.* [50]. Furthermore, the low-frequency coherence between WPO and QBO occurred in autumn (Supporting Information S2). Finally, NEC bifurcation was affected by ENSO, consistent with the results of Qiu and Chen [48]. Note however, most of the signals shown in those wavelet coherence analyses were not significant at *α* = 0.05; thus, these result are just suggestive.

## Discussion

We used long-term glass eel catch data to test the three prevailing hypotheses used to explain the annual recruitment of Japanese eel [15] to Taiwan, subtropical western Pacific: Spawning location hypothesis (H 1), Larval transport hypothesis (H 2), and Ocean condition hypothesis (H 3). H 1 is not supported, as the glass eel catches exhibited a negative relationship with precipitation. This negative correlation is opposite to the hypothesis put forward by Kimura *et al.* [13] and Kimura and Tsukamoto [35]. We are not currently able to explain this finding.

H 2 is plausible. While particle-tracking simulation results suggested that latitudinal shifts in NEC bifurcation critically affect the strength of the Japanese eel larval transport [34], [37], our time series analysis only partially supported this direct transport hypothesis. The catches were correlated with NEC bifurcation with a one-year lag (Table 1) and the wavelet coherence analysis indicated catches often lagged the NEC at different time scales (Figure 4b). Considering the time for larval transport from spawning to Taiwan takes only four to six months [31], the lagged correlation did not support the direct transport hypothesis. However, we cannot rule out the possible error associated with the physical model and/or the estimation of transport time of Japanese eel from spawning to Taiwan. Nevertheless, the significant correlation suggests that latitudinal shifts in NEC bifurcation indeed affected the annual Japanese eel recruitment in some ways. We speculate that the NEC bifurcation index computed here might be indicative of oceanic conditions along the migration route of eel larvae, which may have lagged effects on their recruitment.

Another possible transport effect is the strength (volume) of transport of NEC and Kuroshio toward the western tropical Pacific, but we did not examine this due to lack of data. Qiu and Lukas [36] suggested that the NEC and Kuroshio upstream transport was affected by quasi-biennial changes in surface stress curl, which was modulated by ENSO. Shen *et al.* [64] also found that the long-term Kuroshio transport east of Taiwan indeed reflects the combination of both tropical and extra-tropical climate signals at certain lags: ENSO at a lag of two to four months and WPO at a lag of nine to10 months, respectively. This indicated both tropical and extra-tropical climate impacts should have some delayed influences on Kuroshio transport, which may be the major cause of the one-year lag. However, no direct link has been established here. Interestingly, a study on sea surface temperature (SST) around the south-west of Taiwan revealed a dominant periodicity of 2.6 years (Figure 6b in [8]). This periodicity is very close to the high-frequency fluctuation (2.7 years) observed in the eel catches (Figure 3a) and Niño3.4 (Figure 3d). The dominant peaks in the SST fields south-west of Taiwan may be a local response of ENSO through the propagation of Kuroshio.

We also identified a significant correlation between the catches and QBO (Table 1 and Figure 4d). QBO is a mid-layer atmospheric wind pattern, whose relationship with equatorial surface wind curl is not clear [50]. We are not certain whether or not the QBO affects the NEC and/or Kuroshio transport strength. It is known that the QBO also affects extra-tropic atmospheric dynamics [50] and is modulated by ENSO (Figure 5f); however, how QBO might have an influence on ocean dynamics and the annual eel recruitment remains elusive.

Hypothesis 3 is supported, however indirectly. Significant correlations (Table 1 and Figures 4e–i) are found between catches and climate indices that affect ocean productivity and eddy activities, such as NPGO, PDO, NPI, and WPO. The ecosystem effects of PDO, NPI, and NPGO have been demonstrated in the high and mid-latitude Northeast Pacific and high latitudes of the Northwest Pacific [1], [6], [8], [12], [65], [66]. By contrast, how these climate patterns might have altered the mid latitude Northwest Pacific is relatively unexplored. As suggested by Miller *et al.* [15], these extra-tropic climate patterns may play a critical role in affecting ocean productivities and eddy activities, which in turn would impact on the annual recruitment of the Japanese eel. Analogously, the effects of NAO (extra-tropic climate index in the Atlantic) on the recruitment of European eel have also been proposed [11], [67], [68]. Here, we provide the first empirical analysis for a possible linkage between the extra-tropic climate and the annual recruitment of Japanese eel. We acknowledge, however, that our correlative analyses are suggestive, and further studies of ocean dynamics are needed, perhaps aided by satellite and ocean modeling approaches.

In addition to the PDO, NPGO, and NPI, we found a significant correlation of WPO (Table 1, Figures 1, 3h and 3i). The WPO affects the mesoscale eddy field along the North Pacific Subtropical Countercurrent between 18°–25°N in the western North Pacific, at ∼22°N east of Taiwan [56]. It is possible that the WPO affects the eddy activities (and thus transport) in the region of upstream Kuroshio east of Taiwan [64], which impacts on the annual eel recruitment. To further investigate this possibility, we correlated the catch data with the eddy kinetic energy calculated by Qiu and Chen [56] (data only available after 1992). There is only a suggestion of correlation (r = 0.316, p>0.1). The lack of significant correlation may be due to insufficient data.

While our hypotheses have centered around climatic forcing on larval transport and survival, we should not exclude possible spawner effects [21], [69]. Considering that the Japanese eel spends most of their life in extra-tropic environments and undergo long-distance migration to spawning sites, it is likely the climatic correlations observed here may be explained by variation of adult stock size and, indeed, effects of spawners on recruitment have been suggested in Japanese eel [21].

In addition to the climate indices, we found significant correlation between eel catches and solar activities (Table 1), with coherence at the scale of 11 years (Figure 4j). Our analyses suggested that sunspot numbers modulate the variability of the WPO (Figure 5a). Indeed, previous studies show that the response of climate to solar variability is strongest at mid-latitudes (near 40°), in the vicinity of the interface of the Hadley and Polar cells [70], [71]. While the incoming solar forcing maximizes in zonal bands that track the annually varying subpolar point, climate responds to variations in this energy with maximum warming at mid-latitudes, especially over the North Pacific [72]. According to current understanding, the solar variability could modulate tropospheric circulation patterns through stratospheric–tropospheric radiative and dynamical coupling (via ozone heating) [71]. During high solar activity, the primary meridional circulation in the troposphere weakens in strength and expands in latitude, resulting from the reduced uplift at the equator related to a warmer stratosphere that stabilizes the atmosphere's temperature profile [72]. This could constrain the meridional shift of the dipole associated with the WPO at low frequencies, since the spatial pattern associated with the WPO is a primary mode of the low-frequency atmospheric variability characterized by a north–south dipole of sea level pressure anomalies over the western North Pacific [54]. Nevertheless, the effects of solar activities on the WPO are still not clear. Meehl *et al.* [57] also point out that the enhanced radiative heating in the mid-latitude cloud-free zones during high-solar activity may alter lower latitude moisture, temperature and rainfall. Moreover, QBO has also been shown to relate to the 11-year cycle of solar activities [49], [73].

In fact, an 11-year cycle of correspondence between populations and solar activities has also been observed in terrestrial [74], [75], [76] and marine [74], [75] ecosystems. In terrestrial ecosystems, the solar effects often modulate temperature and/or precipitation, which in turn influence population cycles. However, in marine ecosystems the mechanisms behind the 11-year population cycle remain obscure. Recent studies suggest solar activities may have important effects on climate (see review by [38]). Our study suggested a potential linkage between the ∼11-year cycle of solar activities and the annual recruitment of Japanese eel through WPO. However, note that the coherence of sunspots numbers and WPO is not stationary, with coherence occuring only since the late 1970s (Figure 5a).

For the past decade, scientific research into the Japanese eel recruitment has concentrated on equatorial ocean dynamics. However, the best multivariate regression model includes only the WPO and PDO; both are extra-tropical climate indices. Therefore, our analyses differ by suggesting that extra-tropical climate may play a more important role in influencing the annual recruitment of Japanese eel. Bonhommeau *et al.* [24] used SST as a proxy for ocean productivity, showing that a declining trend of glass eel catches reported from Japan was significantly correlated with a trend of increasing SST (after removing interannual fluctuations using a five-year moving average). We investigated whether this SST of the spawning area of Japanese eel (data from 1967 to 1999 extracted from Bonhommeau *et al.* [24]) affected our annual eel recruitment index, but found no significant correlation (r = 0.175, p>0.3). Even after removing the long-term trend of increasing SST, the correlation remained non-significant (r = 0.027, p>0.8). Thus, the SST of the spawning area is not an important factor for our recruitment data.

Moreover, the dynamics of Taiwanese glass eel catches are not consistent with that of Japanese catches (data from [18]) for both long-term trend and interannual variation (Supporting Information S3). While a long-term declining trend was observed in the Japanese catches, such a declining trend was not so dramatic in the Taiwanese catches. The discrepancy between the annual recruitment of the Taiwanese and Japanese data suggests that additional environmental effects may occur during the Kuroshio transport between Taiwan and Japan. Our findings call for investigation of the mid-latitude ocean dynamics that are likely to affect the survival and transport of glass eels between Taiwan and Japan, in addition to existing research efforts in the equatorial system.

Finally, we should caveat that our analyses were based on glass eel catch data. However, we are certain that the fluctuations and periodicities of eel annual recruitment are not caused by economic forces (Supporting Information S4). While with uncertainty, catch data may still provide useful information to investigate dynamics of exploited stocks in a long-term scale (e.g. [12], [24], [35], [77], [78]).

In summary, using long-term (1967–2008) glass eel catch data, we show that some climate variations are correlated with the catches, suggesting that these environmental effects might have affected the annual recruitment of Japanese eel to Taiwan, subtropical western Pacific. Our results indicate several potential climatic effects on the recruitment. Firstly, variation in NEC might affect the equatorial ocean conditions, which in turn affects larval survival. Again, our analyses do not support the direct transport effect of the NEC bifurcation location, as suggested by a particle-tracking simulation. Secondly, the QBO may influence the NEC transport and/or extra-tropical ocean conditions, which in turn affect larval transport and survival. However, according to Baldwin *et al.* [50], the QBO is less likely to affect the NEC transport. Thirdly, the PDO, NPGO, and NPI may affect mid-latitude ocean conditions, which affected larval survival. Finally, WPO may affect the ocean conditions east of the Philippines and Taiwan through its influence on eddy fields [56], which also affected larval survival. Moreover, WPO and QBO may be modulated by solar activities. These potential mechanisms are not mutually exclusive, because various climate forcing interact at various scales [8]. The potential mechanisms proposed here are suggestive, as one can found that the explained variane is however small in our regression analyses. It is not surprising to see complex environmental effects on biological populations. Fluctuations of the Japanese eel recruitment might not simply be determined by any single environmental factor; rather, those ups and downs may be determined by nonlinear combination of several environmental factors [79], [80]. More detailed studies concerning ocean dynamics and ocean-atmosphere interactions as well as how these factors jointly affect eel recruitment are needed.

The annual recruitment of the Japanese eel to Taiwan might have been affected by multi-scale climatic forcing. We found three periodicities of eel catches (2.7, 5.4, and 10.3 years) and suggested their potential linkage to different climate patterns (Figures 2, 3, and 4). However, these relationships were not stationary (Figures 3 and 4). In fact, a nonstationary nature of ecosystem dynamics may be the norm rather than an exception [14], [61], [62], [81], [82], [83]. Again, the fluctuations in annual eel recruitment may be driven by nonlinear interaction of various climatic factors [79], [80]. Moreover, we observed a long-term declining trend in Taiwanese glass eel catches, although not so marked as that shown in the Japanese catch data. Whether this decline is due to anthropogenic factors requires particular attention. Considering that fishing can elevate the sensitivity of fish distribution to climate [84], better management of glass eel fisheries is needed to mitigate potential overfishing.

## Supporting Information

Supporting Information S1 Results of regression analyses of log_10_(eel catch) against climate indices.
(DOC)

Supporting Information S2 Cross wavelet coherence between climate indices.
(DOC)

Supporting Information S3 Comparison of time series of Taiwanese and Japanese glass eel catches.
(DOC)

Supporting Information S4 The relationship between annual Japanese glass eel catch data and prices.
(DOC)
