# Supplementary material for: Evaluation of Multi-Scale Climate Effects on Annual Recruitment Levels of the Japanese Eel, Anguilla japonica, to Taiwan
Source: PLoS One. 2012 Feb 23;7(2):e30805. doi: 10.1371/journal.pone.0030805 (PMC3285622; doi:10.1371/journal.pone.0030805)
Supplement: Supporting Information S1 — Results of regression analyses of log10(eel catch) against climate indices. (DOC) [file pone.0030805.s001.doc]

**S1. Results of regression analyses of log_10_(eel catch) against climate indices.**

The annual mean was calculated by averaging the four seasons defined here. Note that the year for climate indices starts from spring. Spring: Mar, Apr, May; summer: Jun, Jul, Aug; autumn: Sep. Oct. Nov; winter: Dec and next year Jan and Feb. Data are normalized to unit mean and variance before analyses. Autocorrelation is accounted for using estimated generalized least squares (EGLS). As this univariate analysis is used for exploring potential climate effects, we did not correct for multiple tests. ** indicates that the coefficient is significant at p<0.05; * indicates that the coefficient is significant at 0.05<p<0.1. EKE: Eddy Kinetic Energy (data only available after 1993).

Table S1.1. Regression coefficients of log_10_(eel catch) against climate indices.

|  | Annual | Spring | Summer | Autumn | Winter |
| --- | --- | --- | --- | --- | --- |
| Sunspot | 0.328** | 0.309** | 0.309** | 0.269 | 0.367** |
| NEC | 0.078 | -0.068 | -0.003 | 0.169 | 0.126 |
| Precipitation | -0.132 | -0.047 | -0.235 | -0.019 | 0.071 |
| NINO3.4 | -0.163 | -0.019 | -0.245* | -0.203 | -0.084 |
| NINO4 | -0.171 | -0.069 | -0.221 | -0.185 | -0.119 |
| NINO3 | -0.157 | -0.025 | -0.198 | -0.194 | -0.098 |
| NINO1.2 | -0.068 | 0.019 | -0.041 | -0.072 | -0.143 |
| SOI | 0.045 | 0.037 | 0.048 | 0.048 | 0.014 |
| NPGO | -0.041 | -0.082 | -0.036 | 0.014 | -0.040 |
| PDO | -0.146 | -0.011 | -0.177 | -0.109 | -0.175 |
| NPI | 0.163 | -0.078 | 0.064 | -0.094 | 0.266* |
| QBO | -0.128 | 0.139 | -0.072 | -0.184 | -0.320** |
| WPO | -0.002 | 0.099 | 0.062 | -0.098 | -0.098 |
| EKE | -0.187 | 0.050 | -0.281 | -0.287 | -0.127 |

Table S1.2. Regression coefficients of log_10_(eel catch) against climate indices. Climate index leads eel catch 1 year.

|  | Annual | Spring | Summer | Autumn | Winter |
| --- | --- | --- | --- | --- | --- |
| Sunspot | 0.243 | 0.263 | 0.214 | 0.244 | 0.222 |
| NEC | 0.208 | 0.154 | 0.285** | 0.220 | 0.095 |
| Precipitation | -0.234 | 0.025 | 0.039 | -0.241 | -0.316** |
| NINO3.4 | -0.088 | 0.036 | -0.063 | -0.135 | -0.093 |
| NINO4 | -0.050 | -0.032 | -0.051 | -0.107 | 0.007 |
| NINO3 | -0.147 | -0.019 | -0.153 | -0.164 | -0.138 |
| NINO1.2 | -0.188 | -0.131 | -0.203 | -0.145 | -0.145 |
| SOI | -0.032 | 0.178 | -0.084 | -0.118 | -0.050 |
| NPGO | -0.262 | -0.191 | -0.203 | -0.223 | -0.334** |
| PDO | -0.308** | -0.220 | -0.202 | -0.131 | -0.449** |
| NPI | 0.259 | -0.027 | -0.033 | 0.160 | 0.291** |
| QBO | -0.134 | -0.339** | -0.155 | -0.062 | 0.147 |
| WPO | 0.207 | -0.231 | 0.471** | 0.417** | -0.191 |
| EKE | 0.180 | 0.206 | 0.060 | 0.129 | 0.316 |

Table S1.3. Regression coefficients of log_10_(eel catch) against climate indices. Climate index leads eel catch 2 years.

|  | Annual | Spring | Summer | Autumn | Winter |
| --- | --- | --- | --- | --- | --- |
| Sunspot | 0.167 | 0.194 | 0.198 | 0.100 | 0.159 |
| NEC | -0.180 | -0.097 | -0.235 | -0.170 | -0.135 |
| Precipitation | -0.009 | 0.168 | -0.061 | -0.015 | -0.098 |
| NINO3.4 | -0.006 | -0.087 | 0.000 | -0.012 | 0.040 |
| NINO4 | -0.042 | 0.022 | -0.052 | -0.089 | -0.021 |
| NINO3 | 0.004 | -0.083 | 0.042 | 0.005 | 0.023 |
| NINO1.2 | 0.031 | 0.027 | 0.079 | -0.015 | 0.000 |
| SOI | 0.103 | 0.031 | 0.130 | 0.139 | 0.044 |
| NPGO | -0.158 | -0.116 | -0.148 | -0.221 | -0.108 |
| PDO | -0.224 | -0.399** | -0.212 | 0.032 | -0.138 |
| NPI | -0.080 | -0.083 | 0.182 | -0.068 | -0.074 |
| QBO | 0.224 | 0.394** | 0.256* | 0.105 | -0.053 |
| WPO | -0.289** | 0.063 | -0.207 | -0.274* | -0.269* |
| EKE | 0.188 | 0.002 | 0.079 | 0.330 | 0.211 |

Table S1.4. Regression coefficients of log_10_(eel catch) against climate indices. Climate index leads eel catch 3 years.

|  | Annual | Spring | Summer | Autumn | Winter |
| --- | --- | --- | --- | --- | --- |
| Sunspot | -0.027 | 0.038 | -0.044 | 0.024 | -0.113 |
| NEC | -0.310** | -0.246 | -0.343** | -0.257 | -0.258 |
| Precipitation | -0.048 | -0.068 | -0.216 | -0.106 | 0.267* |
| NINO3.4 | 0.123 | 0.052 | 0.070 | 0.116 | 0.150 |
| NINO4 | 0.086 | 0.078 | 0.042 | 0.057 | 0.109 |
| NINO3 | 0.108 | 0.011 | 0.058 | 0.116 | 0.145 |
| NINO1.2 | 0.101 | 0.082 | 0.082 | 0.109 | 0.061 |
| SOI | -0.147 | -0.154 | -0.160 | -0.165 | -0.033 |
| NPGO | -0.051 | -0.149 | -0.088 | 0.046 | 0.015 |
| PDO | -0.111 | -0.214 | -0.155 | 0.042 | -0.070 |
| NPI | -0.028 | 0.012 | 0.066 | -0.147 | 0.009 |
| QBO | -0.271** | -0.263* | -0.287** | -0.219 | -0.072 |
| WPO | 0.030 | 0.113 | 0.034 | -0.008 | -0.114 |
| EKE | 0.044 | 0.099 | 0.011 | 0.015 | 0.071 |

Table S1.5. Regression coefficients of log_10_(eel catch) against climate indices. Climate index leads eel catch 4 years.

|  | Annual | Spring | Summer | Autumn | Winter |
| --- | --- | --- | --- | --- | --- |
| Sunspot | -0.184 | -0.134 | -0.188 | -0.173 | -0.208 |
| NEC | -0.135 | -0.249 | -0.109 | -0.108 | -0.026 |
| Precipitation | 0.038 | 0.005 | 0.076 | -0.017 | 0.016 |
| NINO3.4 | -0.046 | 0.107 | -0.043 | -0.075 | -0.087 |
| NINO4 | -0.095 | 0.113 | -0.079 | -0.182 | -0.137 |
| NINO3 | -0.046 | 0.054 | -0.069 | -0.057 | -0.054 |
| NINO1.2 | -0.072 | -0.002 | -0.089 | -0.058 | -0.090 |
| SOI | -0.037 | -0.022 | 0.024 | -0.031 | -0.070 |
| NPGO | -0.127 | -0.129 | -0.070 | -0.141 | -0.132 |
| PDO | -0.083 | -0.189 | -0.008 | 0.051 | -0.128 |
| NPI | 0.055 | 0.077 | -0.158 | 0.175 | -0.014 |
| QBO | 0.331** | 0.102 | 0.313** | 0.362** | 0.247* |
| WPO | 0.102 | 0.271* | -0.020 | 0.114 | -0.225 |
| EKE | 0.058 | 0.022 | 0.007 | 0.093 | 0.087 |

Table S1.6. Regression coefficients of log_10_(eel catch) against climate indices. Climate index leads eel catch 5 years.

|  | Annual | Spring | Summer | Autumn | Winter |
| --- | --- | --- | --- | --- | --- |
| Sunspot | -0.158 | -0.203 | -0.095 | -0.171 | -0.144 |
| NEC | 0.052 | -0.069 | 0.038 | 0.071 | 0.123 |
| Precipitation | -0.034 | -0.040 | -0.093 | 0.096 | -0.026 |
| NINO3.4 | -0.074 | -0.154 | -0.117 | -0.058 | 0.006 |
| NINO4 | -0.206 | -0.187 | -0.228 | -0.215 | -0.126 |
| NINO3 | -0.014 | -0.099 | 0.013 | -0.019 | 0.024 |
| NINO1.2 | 0.033 | -0.074 | 0.104 | 0.006 | 0.062 |
| SOI | 0.163 | 0.263 | 0.086 | 0.089 | 0.100 |
| NPGO | -0.051 | -0.099 | -0.030 | -0.091 | 0.025 |
| PDO | -0.047 | 0.009 | -0.022 | -0.106 | -0.032 |
| NPI | -0.057 | -0.167 | -0.008 | 0.080 | -0.018 |
| QBO | -0.283* | 0.007 | -0.237 | -0.330** | -0.345** |
| WPO | -0.196 | -0.182 | 0.150 | -0.142 | -0.236 |
| EKE | 0.222 | 0.288 | 0.113 | 0.177 | 0.314 |
