# Supplementary material for: Evaluation of Multi-Scale Climate Effects on Annual Recruitment Levels of the Japanese Eel, Anguilla japonica, to Taiwan
Source: PLoS One. 2012 Feb 23;7(2):e30805. doi: 10.1371/journal.pone.0030805 (PMC3285622; doi:10.1371/journal.pone.0030805)
Supplement: Supporting Information S2 — Cross wavelet coherence between climate indices. (DOC) [file pone.0030805.s002.doc]

**S2. Cross wavelet coherence between climate indices**


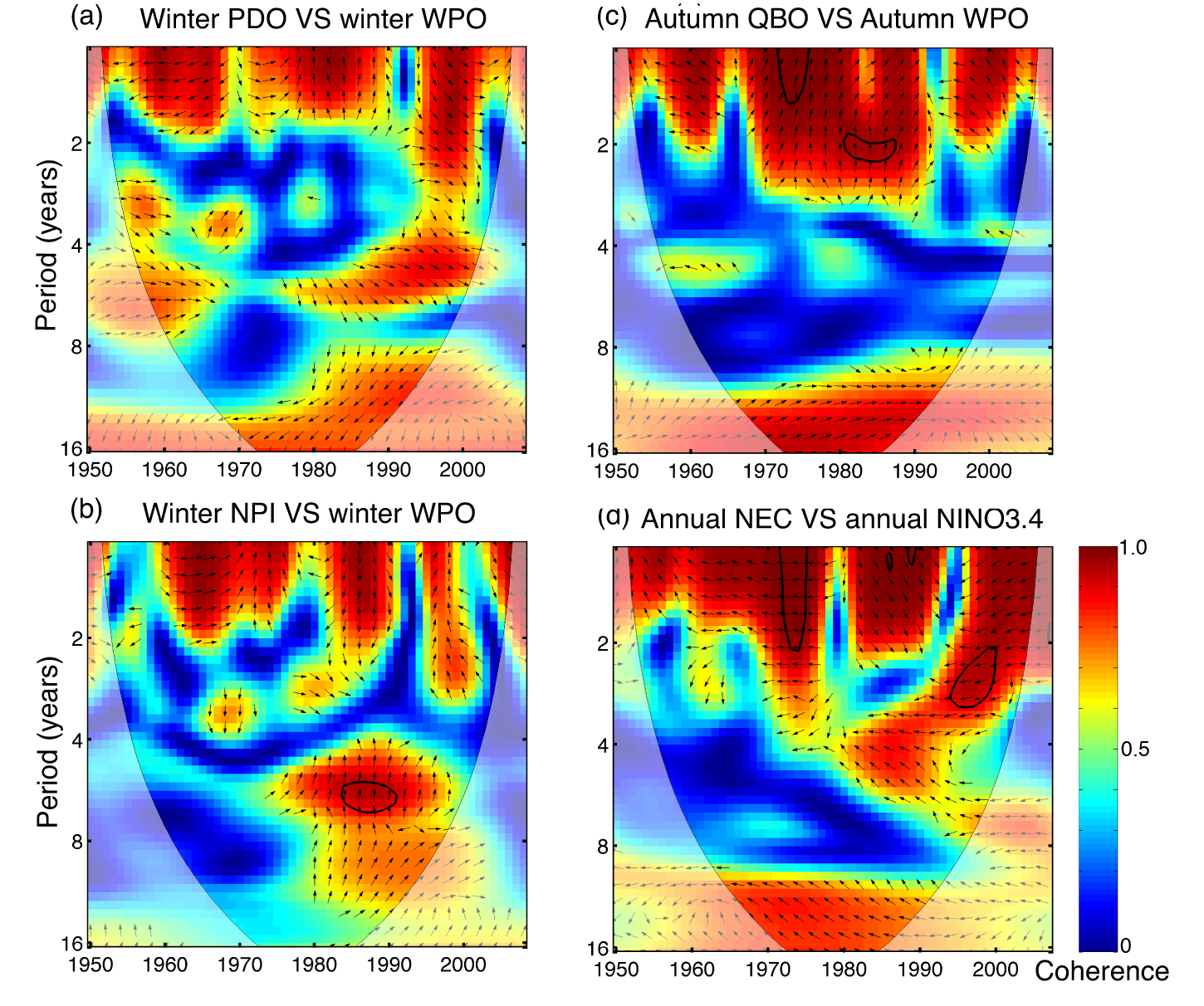


Figure S2.1. Cross wavelet coherence between climate indexes: (a) winter PDO versus WPO, (b) winter NPI versus WPO, (c) autumn QBO versus WPO, and (d) annual NEC versus Niño3.4. The solid black contour encloses regions of greater than 95% confidence, and the shadowed area indicates the cone of influence where edge effects become important. The phase relationship is shown as arrows, with in-phase pointing right, anti-face pointing left, and former leading the later index (as shown in the title of each panel) by 90° pointing straight up.
