# Supplementary material for: Evaluation of Multi-Scale Climate Effects on Annual Recruitment Levels of the Japanese Eel, Anguilla japonica, to Taiwan
Source: PLoS One. 2012 Feb 23;7(2):e30805. doi: 10.1371/journal.pone.0030805 (PMC3285622; doi:10.1371/journal.pone.0030805)
Supplement: Supporting Information S3 — Comparison of time series of Taiwanese and Japanese glass eel catches. (DOC) [file pone.0030805.s003.doc]

**S3. Comparison of time series of Taiwanese and Japanese glass eel catches.**


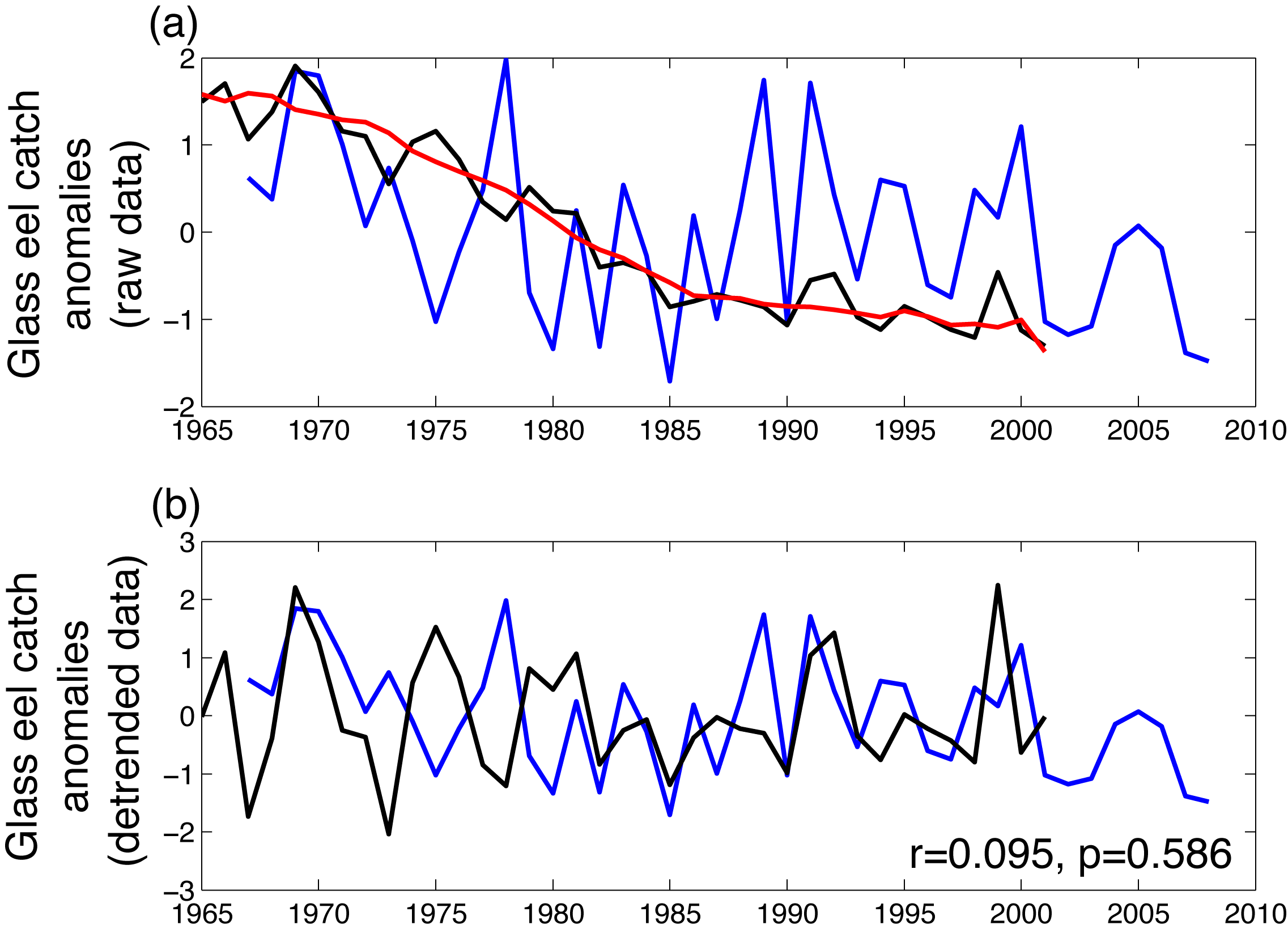


Figure S3.1. Comparison of time series of Taiwanese (blue) and Japanese (black) glass eel catches. Time series are log10-transformed and normalized to unit mean and variance. The red line represents the long-term trend of the Japanese catches computed used LOWESS with a span of 10 years. In (a), plotted are anomalies of raw data. In (b), plotted are anomalies of raw data for the Taiwanese catches (because no significant long-term trend exists) and residuals deviated from the trend (red line) for the Japanese catches. No significant correlation exists between the interannual variation of Taiwanese and Japanese glass eel catches. Even considering only the later period (1980 to 2001), the correlation remains not significant (r=0.283, p>0.2). Our conclusion is not sensitive to the span of LOWESS. Sensitivity analyses on span of 5, 10, 15, 20, and 30 years show the same conclusion.
