# Supplementary material for: Evaluation of Multi-Scale Climate Effects on Annual Recruitment Levels of the Japanese Eel, Anguilla japonica, to Taiwan
Source: PLoS One. 2012 Feb 23;7(2):e30805. doi: 10.1371/journal.pone.0030805 (PMC3285622; doi:10.1371/journal.pone.0030805)
Supplement: Supporting Information S4 — The relationship between annual Japanese glass eel catch data and prices. (DOC) [file pone.0030805.s004.doc]

**S4. The relationship between annual Japanese glass eel catch data and prices.**

We collected the annual price data of Japanese glass eel in Taiwan. The economic inflation in price was corrected using the Consumer Price Index of Taiwan. There is a marginal negative relationship between catches and prices (r=-0.2469, p=0.115), but this relationship is not statistically significant. As can be seen in Figure D1, the negative correlation between catches and prices became clearer after 1980. It is intuitively to consider that high catches could result in a lower price in that year, but it is difficult to imagine a high price could result in low catches. Thus, it can be assumed that the catches may determine the prices but not the reverse.

We also tested the causal relationship between catches and prices using the vector auto-regression model (VAR) with lags up to three years. To meet the assumption of VAR, time series were first differenced. Prior to the analysis, the time series were normalized to unit mean and variance. The results of VAR indicate no clear causal relationship between the catches and prices, while self auto-regression was significant in catch data (Table D1). Therefore, the fluctuations and periodicities of Japanese eel catch data are not caused by economic forces.


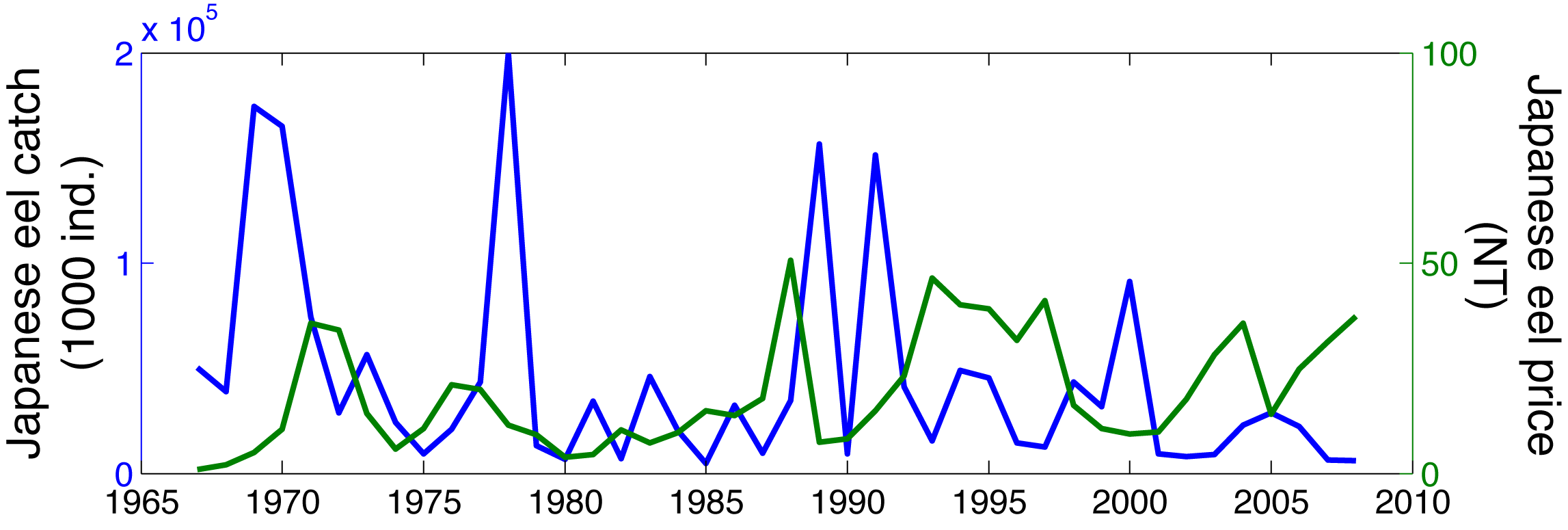


Figure S4.1. Time series of glass eel catch data (blue line) and prices after correcting the economic inflation (green line).

Table S4.1. Results of the test of causality based on the vector auto-regression model. Results indicate that catches do not cause prices, and prices do not cause catches.

| Response variable | Forcing variable | Lag coefficient | *t*-statistic | p-value |
| --- | --- | --- | --- | --- |
| Catch(t) | Catch(t-1) | -0.5443 | -3.0627 | 0.0045 |
| Catch(t) | Catch(t-2) | -0.4173 | -2.3341 | 0.0262 |
| Catch(t) | Catch(t-3) | -0.1806 | -1.1470 | 0.2602 |
| Catch(t) | Price(t-1) | 0.2167 | 1.4902 | 0.1463 |
| Catch(t) | Price(t-2) | -0.1196 | -0.8357 | 0.4097 |
| Catch(t) | Price(t-3) | 0.2010 | 1.3871 | 0.1753 |
| Price(t) | Catch(t-1) | -0.1125 | -0.5049 | 0.6172 |
| Price(t) | Catch(t-2) | 0.2684 | 1.1971 | 0.2404 |
| Price(t) | Catch(t-3) | -0.0389 | -0.1972 | 0.8450 |
| Price(t) | Price(t-1) | -0.2542 | -1.3939 | 0.1733 |
| Price(t) | Price(t-2) | -0.1562 | -0.8704 | 0.3908 |
| Price(t) | Price(t-3) | -0.3384 | -1.8621 | 0.0721 |

**Supporting references**

1. Watson MW (1994) Vector autoregressions and cointegration. In: Engle RF, McFadden DL, editors. Handbooks of Economics. pp. 2844-2915.
